# To: Risk factors, impact on outcomes, and molecular epidemiology of infections caused by carbapenem-resistant *Enterobacterales* in intensive care patients: a multicenter matched case-control study in Brazil

**DOI:** 10.62675/2965-2774.20250177

**Published:** 2025-11-03

**Authors:** Hinpetch Daungsupawong, Viroj Wiwanitkit

**Affiliations:** 1 Private Academic Consultant - Phonhong Lao People's Democratic Republic Private Academic Consultant - Phonhong, Lao People's Democratic Republic.; 2 Dr. D Y Patil Vidyapeeth (Deemed to be University) Hospital and Research Centre Department of Community Medicine Pune India Department of Community Medicine, D Y Patil Medical College, Hospital and Research Centre, Dr. D Y Patil Vidyapeeth (Deemed to be University) - Pune, India.

## TO THE EDITOR

The publication "Risk factors, impact on outcomes, and molecular epidemiology of infections caused by carbapenem-resistant *Enterobacterales* in intensive care patients: a multicenter matched case-control study in Brazil"^(1)^ is hereby discussed. The purpose of this study was to look at risk factors, genetic traits, and hospital death rates in critically ill patients who had carbapenem-resistant *Enterobacterales* (CRE) infection. The study's merits include a multicenter cohort design and the use of propensity score matching, which lowers bias to some extent. However, the retrospective methodology remains limited due to inadequate data or bias from retrospective data recording. Despite its benefits in patient matching, a layered case-control design may limit the capacity to infer causality. Furthermore, utilizing only 1:1 matching may result in the loss of valuable samples, lowering the statistical power of the research.

Conditional and multivariable logistic regression are statistically sound options for analyzing risk variables and outcomes, but further information, such as assessing multicollinearity across covariates and examining model fit, is required. Furthermore, the large confidence intervals for some risk factors, such as premorbid assistance (OR 1.72, 95%CI 0.99 - 3.01), suggest that the conclusions are unclear. This could be due to insufficient sample sizes for specific subgroups. Bootstrapping or sensitivity analysis is recommended to increase confidence in the results.

The potential processes between CRE infection with coronavirus disease 2019 (COVID-19) and the combination of multiple risk factors, such as the use of broad-spectrum antibiotics or the frequency of invasive procedures, that may lead to drug-resistant infections should be discussed further. Furthermore, research should consider the influence of unique ICU features, which may include varying infection control programs and antibiotic stewardship. The time from infection to death has not been described, which may help to distinguish between causes of mortality directly connected to CRE.

Future research directions should include prospective cohort studies to examine the causal relationship between risk factors and death and omics technologies such as transcriptomics or proteomics to better understand the body's response to CRE at the molecular level and add novelty to the study. Furthermore, investigations on the effects of infection prevention measures such as hand washing, antibiotic limitation, and isolation of drug-resistant patients are critical concerns for developing concrete guidelines.
